# Effect of platelet rich plasma injection on bone formation in the expanded mid-palatal suture in rabbits: a randomized controlled animal study

**DOI:** 10.1186/s12903-024-03922-6

**Published:** 2024-02-02

**Authors:** Sherief H. Abdel-Haffiez, Nesma Mohamed Khalil

**Affiliations:** Alexandria University, Alexandria, Egypt

**Keywords:** Platelet rich plasma, Rabbits, Histomorphometry, Immunohistochemistry, Midpalatal suture expansion, Osteopontin, Bone formation, Vascular denisity, Osteoblast count

## Abstract

**Background:**

Mid-Palatal suture expansion needs long retention period due to delayed bone formation in the expanded suture. Platelet-rich plasma (PRP) is a concentrated source of growth factors which increase bone formation. The aim of this study was to evaluate the effect of PRP injection on bone formation in expanded mid palatal suture in rabbits.

**Methods:**

In this prospective randomized controlled animal study, Twenty male rabbits (8-weeks-old) were subjected to mid-palatal expansion for 5 days. Animals were afterwards randomly divided into control group A & study group B. PRP was prepared and injected in the mid-palatal suture in animals belonging to group B only. After 6 weeks of retention, all animals were euthanized, and premaxillae were prepared for histological, histomorphometric and immunohistochemical analysis. Student t-test and paired t-test were used to compare the means of the two groups and within the same group respectively. Significance level set at *p ≤ 0.05*.

**Results:**

Histomorphometric analysis revealed a significant increase (*p <* 0.001) in the mean percentage of new bone in the study group (14.4%) compared to the control (1.4%). Suture width in study group was significantly wider than the control group (278.8 ± 9μms and 120.4 ± 3.4μms, *p <* 0.001). There was a significant increase in vascular density in study group than control group (309 ± 65.34 and 243.86 ± 48.1, *p* = 0.021). Osteopontin immuno-expression revealed a significant increase in optical density in study group than control group (0.21 ± 0.02 & 0.12 ± 0.01, *p <* 0.001).

**Conclusions:**

In rabbit model, PRP injection can accelerate new bone formation in the expanded mid-palatal suture when compared to the control. This could hopefully result in a more stable midpalatal expansion and a reduced retention period.

## Introduction

Maxillary constriction is a relatively common problem in patients seeking orthodontic treatment. Correction of such problem can be performed using an appliance that imposes tension force to separate the two palatal bones from each other at the midpalatal suture and consequently increases the maxillary transversal dimension. During this procedure, tension forces are built in the suture inducing bone remodelling. New bone formation and collagen fiber rearrangement continues as part of sutural bone remodelling until equilibrium is achieved [1–3]. However, early relapse of expanded midpalatal suture has frequently been reported. [4]A major reason for such early relapse has been attributed to inadequate or delayed bone formation in the midpalatal suture as part of the sutural remodelling process following its separation [5].

The early induction of new bone formation in the recently expanded midpalatal suture will result in early stabilization of the expansion process and consequently would reduce the possibility of expansion relapse and shorten the period needed for retention. Different methods have been used in the literature to evaluate the outcome of suture expansion. Suture width is an important parameter used to evaluate the outcome of the suture expansion. Alyessary et al. [6] measured the sutural separation as one of the histomorphometric variables to evaluate mid-palatal suture expansion and Willershausen et al. [7] recorded the suture width as one of the histomorphometrical parameters to analyse hard palate specimens at different ages. Immunohistochemical markers also were used to investigate molecular biology in areas of osteogenesis. CD34 is a surface molecule expressed specifically on hematopoietic cells and on endothelial cells [8] and is used to detect vascularity. Osteopontin is a glycosylated phosphoprotein produced by different types of cells including osteoblasts, osteocytes, and inflammatory cells [9]. It plays a role in bone homeostasis and affects the activity of bone cells [10]. Osteopontin expression in osteoblasts and osteocytes was found to increase in response to mechanical tension like suture expansion [11–14].

Many successful methods to induce faster bone formation in the expanded midpalatal suture have been documented in the literature, including laser therapy [15, 16], lithium chloride [17], vitamins [18, 19], bisphosphonates [20], dietary boron [21] and antioxidants [22, 23]. However, in the era of tissue engineering, a remarkable concern should be given to autologous biological product such as platelet concentrates. This excludes any possibility of side effects that could be associated with the use of chemical substances, and excludes the possibility of cross infections. Compared to the other forementioned methods, platelet concentrates have the superiority for [1] being rich in growth factors that are important for bone healing [24–26], (2) contains variable blood proteins that are important for osteoblasts and fibroblasts chemotaxis [27], (3) considered a potent initiator for bone regeneration and apposition when injected at a local site [27–31].

The use of platelet concentrates as a healing booster in different medical and dental fields has been established since 1998 when Marx came up with the 1st generation platelet concentrate known as Platelet Rich Plasma (PRP) [27]. However, two principal limitations were associated with PRP use [32, 33]. First limitation is the adverse reactions that may affect the host caused by the presence of an external anticoagulant used during PRP preparation to prevent blood coagulation. And the second limitation is the relative rapid release of growth factors from PRP upon activation. To overcome these limitations, Choukron in 2001 [34] introduced the 2nd generation platelet concentrate, Platelet Rich Fibrin (PRF). Because the preparation of PRF does not include an additional anticoagulant, a blood clot is formed consisting of a fibrin matrix entrapping platelets and leukocytes in it. The presence of this 3D fibrin matrix slows and prolongs the release of growth factors in comparison to PRP. PRF showed superior results when combined with graft materials or used to heal bony defects compared to PRP in periodontology, maxillofacial and implant dentistry [35–37].

However, PRF has a gel consistency making it is more suitable to be added to graft materials or to hollow defects and cannot be injected into tissues [38]. To overcome the injection limitations, Choukron introduced the injectable PRF (i-PRF), that can be injected into tissues within 15 minutes before it starts coagulation [39]. However, injectable PRF (i-PRF) did not show the expected superiority over PRP as PRF did. Recent studies showed no difference in the rate of tooth movement during canine retraction [40] or lower incisor alignment [41] when both PRP and i-PRF were compared. A review of randomized controlled trials [42] concluded equal effect of (PRP) and (PRF) on acceleration of orthodontic tooth movement and a recent systematic literature review [43] demonstrated clinical privileges for the use of PRF in periodontology, oral medicine and oral surgery, with unclear benefits in the fields of endodontics and orthodontics. Even in the field of orthopaedics, a recent systematic review [44] demonstrated PRP superiority over i-PRF in arthroscopic rotator cuff repair.

The proved efficacy of PRP in injections and the unproven superiority of i-PRF over PRP could be due to 3 important reasons:

1. Injected PRP is activated when it comes to contact with collagen type I naturally present in the host tissues [45, 46]. This activation pattern has been suggested to activate the PRP and release its growth factors in a slower and sustained manner over time [47].
2. The preparation of i-PRF adopts a low-speed centrifugation technique, lead to the incorporation of white blood cells, monocytes, endothelial cells, stem cells and other various cellular elements in addition to the platelets. This makes i-PRF a blood concentrate rather than a solid pure platelet concentrate [48].
3. Cell concentration in (i-PRF) is not homogeneous, with platelets mainly located at the interface between the yellow and red phase [49].

The literature proved local injection of PRP as a reliable strategy for osteoblasts promotion with the resultant increase in the rate of bone formation, and improving mineralization and density of cancellous bone [27, 50, 51]. Furthermore, PRP proved efficacy in orthodontic tooth movement acceleration [52–54] and successfully reduced tooth movement induced by relapse after orthodontic tooth movement [55].

Therefore, the possibility of using the biologically safe osteoinductive PRP to regenerate bone in the expanded mid-palatal suture may be useful in controlling relapse following expansion. The specific aim of the current study was to evaluate the general histological changes in the expanded mid-palatal suture following PRP injection and to measure the percentage of surface area of newly formed bone, osteoblasts’ count, percentage of unmineralized bone, immunoreactivity to CD34 antibodies and osteopontin associated with PRP injection into expanded mid-palatal suture. The null hypothesis is that platelet rich plasma injection in the mid-palatal suture after expansion will not result in promoting bone formation in the suture.

## Materials and methods

### Study design

In our prospective randomized controlled animal experiment to study the effect of PRP injection on new bone formation in the expanded midpalatal suture, 8-weeks-old New Zealand rabbits were used. The rabbit model was more suitable than a rat model for the current study as a relatively large blood sample suitable for PRP preparation could be withdrawn from the rabbit without deleterious effect on the animal’s general health [56]. In addition, rabbits have haversian system like humans, so the results can be correlated to humans [57]. All rabbits included in the study were chosen to be males due to the prevalence of blood coagulation problems in female rabbits [56]. All included rabbits were chosen within the normal weight suitable for this age (2 kg – 2.5 kg) and with normal activity and behaviour. Any rabbit showed any dermatological lesions or any other signs of systemic or local diseases were excluded from the study.

### Sample size estimation

The minimal sample size was calculated based on a previous similar study aimed to evaluate bone regeneration following mid-palatal suture expansion in rabbits [21]. The minimum required sample size to detect the difference in newly formed bone surface area was found to be ten rabbits per group (number of groups = 2) adopting a power of 80% (β = 0.20) to detect a standardized effect of newly formed bone surface area (primary outcome) of 1.200, at level of significance 5% (α error accepted = 0.05) [58]. Any sample withdraws from the study due to any reason will be replaced to maintain the sample size [59]. The sample size was calculated using GPower version 3.1.9.2 [60].

### Study setting

This study was carried out in accordance with the ARRIVE guidelines for reporting animal research [61]. This experiment followed the guidelines of the Alexandria University Ethics Committee for the Animal Experimentation and was performed after gaining the approval of the Research Ethics Committee of Alexandria University Faculty of Dentistry (IRB No. 00010556 – IORG 0008839).

The experiment was carried out at the animal house of Medical Research Institute, Alexandria University. Animals were kept in the experimental animal house under similar environmental conditions. They were kept in polypropylene cages under 12 hour’s light–dark periods and a temperature (24 ± 2 °C) with free access to water and commercial diet. Histological and histomorphometrical analysis were conducted in the Oral Biology Department, Faculty of Dentistry, Alexandria University.

### Intervention

Each animal was weighted before starting any interventions and the weight of each animal was recorded in kilograms.

### Animal anesthesia [62]

All the rabbits were anesthetized to perform the experimental procedures by intramuscular injection of Ketamine (35 mg/kg) *(Ketamine hydrochloride injection USP, rotex medica, Trittau, Germany)* and Xylazine (5 mg/kg) *(Xyla-Ject, Adwia, 10th of Ramadan City, Egypt)*.

### Appliance fabrication [21]

**Fig. 1:**
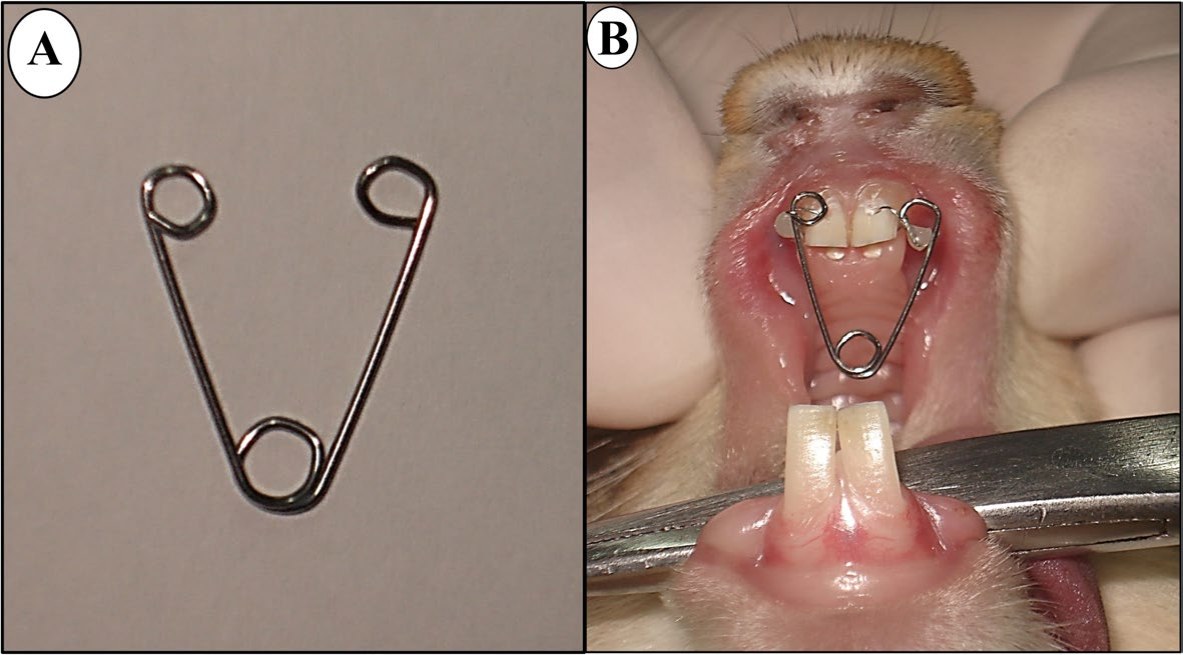
**a** Helical spring expander. **b** Expansion spring in situ

A helical spring fabricated from 0.028-in. stainless steel wires were used for mid-palatal expansion (Fig. 1a). The spring was simple design with no laboratory preparation and easily installed in place. The expanding force were adjusted using a gauge to (250 g). In all the animals, under anaesthesia, each arm of the expansion spring was ligated using a piece of ligature wire to one central incisor into a hole drilled at the level of the lingual gingival papilla (Fig. 1b). The distance between the 2 mesio-incisal angles of the 2 central incisors were measured (T0) using a digital caliper [63].

Active mid-palatal suture expansion was executed in all animals with the expansion spring for 5 days [21]. Each animal was given a number. Given numbers were attached to the rabbit’s ear using a piece of ligature wire. This number was used for randomized allocation in the study groups according to a previously generated table. Randomization table generation and rabbits’ allocation into the groups were done using a computer-generated random list [64]. Afterwards**,** animals were equally and randomly assigned into one of two groups A and B. Group A was assigned a control group with no further interventions following midpalatal suture expansion, whereas in Group B (Study group), PRP was injected in the midpalatal suture following midpalatal expansion.

### PRP preparation and injection

PRP was prepared for each animal assigned to group B at the laboratories of the Department of Clinical Pathology, Faculty of Medicine, Alexandria University, after active mid-palatal suture expansion. To avoid premature platelet activation, a needle gauge (≥ 22) was used in blood sample collection for PRP preparation [46]. Therefore, intracardiac punctures were used for blood sample collection as other puncture sites such as ear or femoral veins were not suitable with the large recommended needle gauge. Blood (10 ml) was drawn from each animal to 10% sodium citrate preloaded syring to avoid adverse effects on platelet membrane viability associated with other anticoagulants as EDTA and heparin [65].

Autologous PRP was prepared using the double spin technique [66, 67]. The blood samples underwent the first centrifugation for 10 minutes at 160 G and the middle and top layers were extracted and transferred to a new tube. The samples were then centrifuged for the second round at 400 G for 15 minutes to produce PRP in the lower third of the tube.

Animals were anesthetized again. The distance between the mesio-incisal angles of both right and left central incisors were measured (T1) to assess the amount of expansion attained. An occlusal x-ray was obtained for all the animals using a size 2 periapical film to verify mid-palatal suture opening with the installed appliance. The expansion springs were replaced by a piece of 18*25 stainless steel wire for passive retention of the expansion in all the animals [21]. PRP was injected in the mid-palatal suture in animals belonging to group B using citoject periodontal injector (SOPIRA Citoject syringe, Heraeus Kulzer Inc. South Bend, USA). PRP was injected in the liquid form with no pre-activation as platelet activation would occur upon PRP contact with the tissue collagen [45, 46] resulting in more physiologic activation and prolonged growth factors release from PRP [46, 47].

The marked needle (using an endodontic file rubber stop) was inserted 3 mm into the tissues of the midpalatal suture and 0.7 ml of PRP was injected using the ‘peppering’ technique. This involves inserting the needle into the tissues, injecting some of the PRP withdrawing the needle but without emerging it, slightly redirecting, reinserting and reinject. On the other hand, all the animals in group A were left without any further intervention.

Expansion was retained for 6 weeks in both groups (A and B) as complete bone regeneration in rabbits requires 42 days to take place [6, 68]. After the retention period and before animal euthanizea, each animal was weighted again in kilograms and increase or decrease in each animal’s weight was assessed. Animals in both groups were euthanized by decapitation following the American Veterinary Medical Association guidelines after administration of intraperitoneal sodium pentobarbital (100 mg/kg) *(Pentobarsol, Dechra, Overland Park, KS, USA)* after 6 weeks of retention period and the premaxillae of the sacrificed animals were obtained and prepared for histological examination and histomorphometric analysis.

### Histological evaluation

After animal sacrifice, premaxillae were harvested and cleaned from any soft tissue remnants and debris. Neutral buffered formalin (10%) was used for specimens’ fixation for 48 hours [69].

Afterwards, 5% formic acid was used for decalcification for 5 days (changed 3 times per day) [21]. The specimens were immersed in increasing concentrations of alcohol for dehydration, followed by clearing in xylene and infiltration with paraffin wax. Specimens were finally embedded in paraffin blocks in an orientation that will make the direction of cutting at right angle to the sagittal plane and to pass through gingival part of the crown of incisor at its center and joining 2 points one of them assigned at the crest of alveolar bone and the second point was assigned 4 mm apical to the first point [21]. The sections were cut at a thickness of 5 μm and stained with hematoxylin and eosin stain (H&E) for general examination [69] and Goldner-Masson trichrome stain for detection of the unmineralized newly formed bone [70] and examined by light microscope.

### Data collection

A) ***Histomorphometric analysis***

Histomorphometric analysis was performed using Image J 1.46r (National Institutes of Health, Bethesda, MD), a reliable method used as a quantitative measurement of the new bone formation [71, 72]. The measurements were done in 5 serial sections in every specimen in each group and a mean was calculated. The same procedure was repeated in each of the ten specimens in each group. The following parameters were assessed:

1. **The percentage of new bone surface area.**

**Fig. 2:**
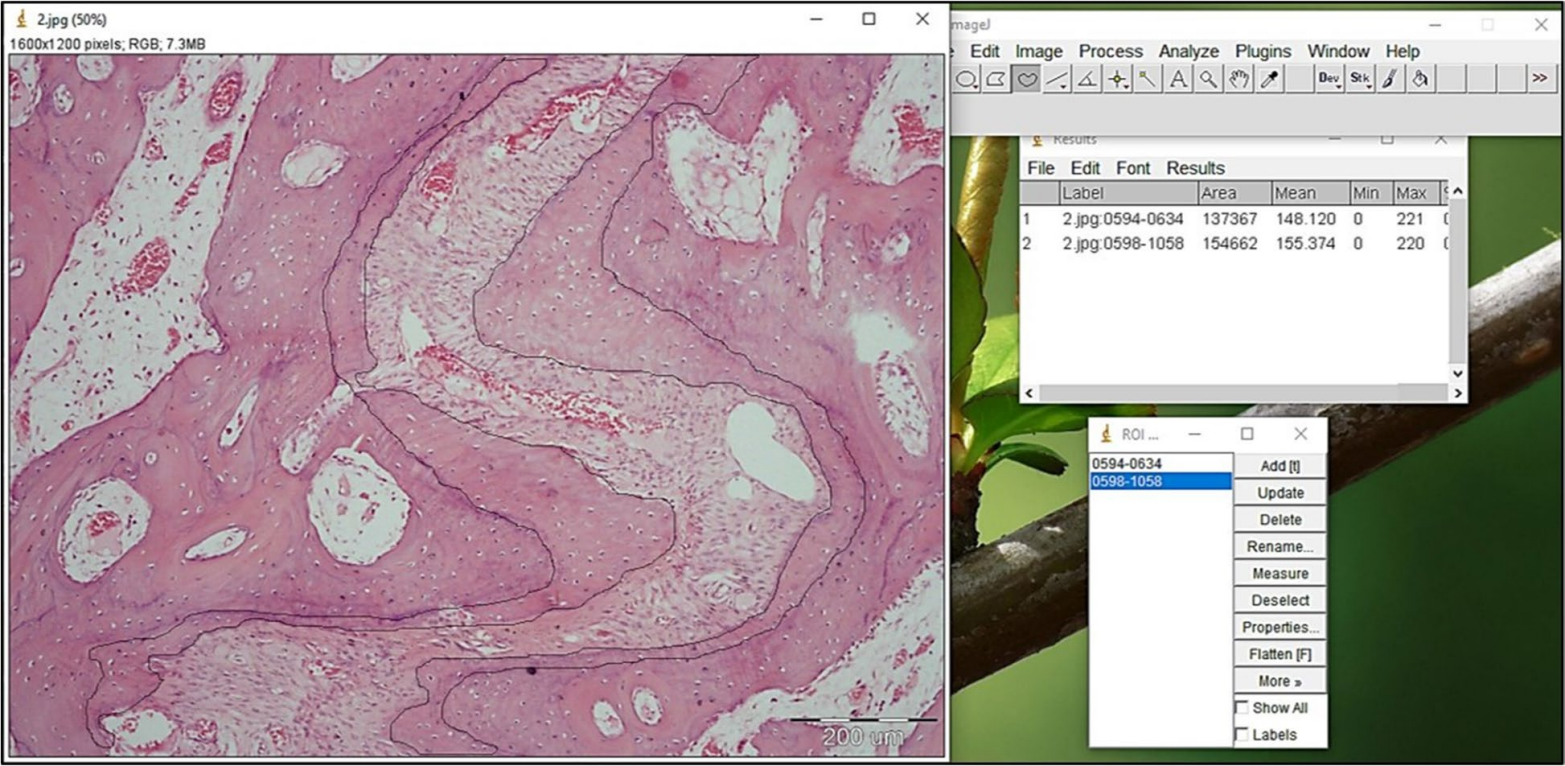
Tracing of newly formed bone using image J

The analysis was carried out on light microscopic pictures with a magnification of × 100. The newly formed bone was traced using free hand selections tool, then its surface area was measured. The percentage of the newly formed bone was calculated in relation to the total surface area of the field (Fig. 2).

2- **Mean suture width of the mid palatal suture** [73].

**Fig. 3:**
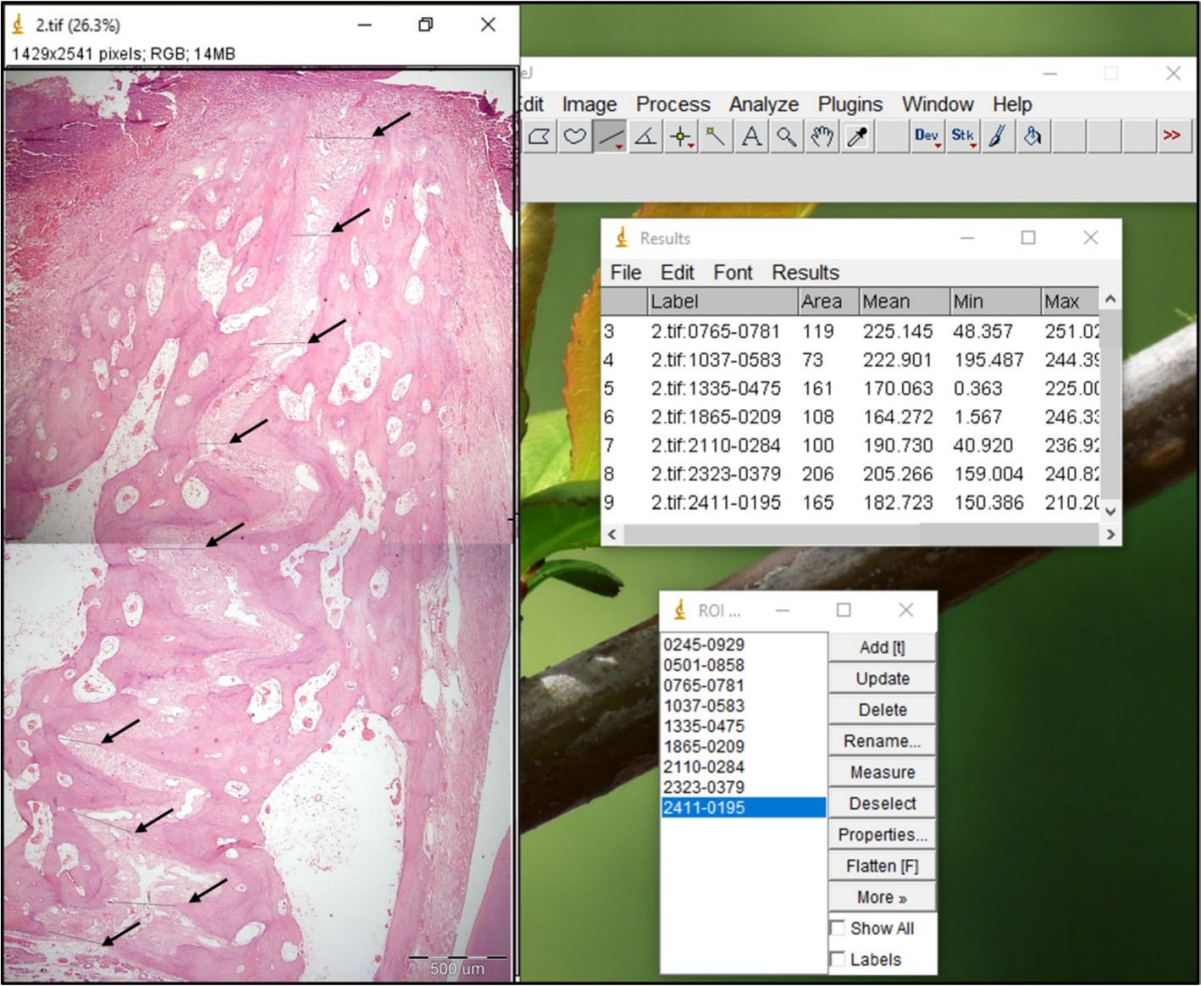
Measurement of mean suture width using image J

The analysis was carried out on light microscopic pictures with a magnification of × 40. A line was drawn between the 2 sides of the suture and it was measured. The same procedure was repeated at 9 different points distributed evenly and equally along the whole length of the suture. Then the mean suture width was obtained. The same procedure was repeated in all the specimens in both control and study groups Fig. 3.

3- **Osteoblasts’ count**

The analysis was carried out on light microscopic pictures with a magnification of × 400. In each section, ten non-overlapping images were used along the whole suture length and the mean was calculated.

4- **Percentage of unmineralized bone**

In Goldner-Masson trichrome stained sections (× 100), the unmineralized bone was stained red and was measured as its percentage to the total surface area of the field.

B) ***Immunohistochemical analysis***

Immunohistochemical evaluation of both CD34 and osteopontin were evaluated in 5 serial sections (5 μ thick) in each specimen. In each section, ten non-overlapping images (× 400) were used along the whole suture length and the mean was calculated.

Sections on silanized slides were first deparaffinized in xylene, rehydrated in graded concentrations of ethanol. For antigen retrieval, citrate buffer solution was used at 100 °C for 20 minutes. To reduce nonspecific binding of antibody, 1% bovine serum albumin was used for half an hour at room temperature in phosphate buffered saline (PBS) solution. Sections were then incubated with primary antibody for 60 minutes. This was followed by washing by buffer and incubation with secondary antibody for half an hour and then washed in PBS. For antibody detection, 3,3′-Diaminobenzidine (DAB) was used. Finally, counterstaing of the sections was accomplished by Mayer’s hematoxylin [6]. Negative control were preapred by using PBS instead of primary antibody.

- **CD34**

To assess the vascularity in the mid-palatal suture, immuno-staining of CD34 (a marker expressed on endothelial cells) was done using anti-CD34 antibodies (Thermo Fisher Scientific, Fremont,CA, USA).

1. **Optical density (OD)** [74]

The optical density of the CD34 positive blood vessels was calculated using the following equation: OD = log (maximum intensity/mean intensity), maximum intensity = 255.

2- **Vascular density** [7]

In each image, the total surface area of the suture was traced and measured, and the positively stained CD34 blood vessels were counted. The vascular density was calculated as number of blood vessels per 1mm^2^ of the suture total surface area.

- **Osteopontin**

Immuno-staining of osteopontin was done using anti-osteopontin antibodies (Medaysis, Livermore, CA, USA). The optical density of osteopontin immune-staining and area percent [74] (the percentage of area stained positive for osteopontin to the total area of the field) were calculated.

### Blinding and intra-examiner reliability

Prepared histological slides’ labels were covered by scotch tape for blinding purposes during histological examination, histomorphometric and immunohistochemical analysis. Comments and measurements were performed by one researcher (K.N) with scotch tape coverings. Also, the statistician was blinded for the groups during data analysis.

After 2 weeks from performing the first measurements, five specimens from each group were randomly selected to repeat the measurements of percentage of new bone surface area, the mean suture width of the mid palatal suture, osteoblasts’ count, percentage of unmineralized bone and immunohistochemical analysis by the same researcher. Intraclass correlation coefficient (ICC) was used to assess the intra-examiner reliability.

### Data analysis

Data was presented as mean ± standard deviation. Data was analyzed using IBM SPSS software package version 20.0. **(**Armonk, NY: IBM Corp**)**. Shapiro-Wilk test revealed normal distribution of the data. Student t-test was used to compare the means of two groups for normally distributed quantitative variables and paired t-test was used to compare the means within the same group. Significance of the obtained results was judged at the 5% level.

## Results

The value of Intraclass correlation coefficient (ICC) was 0.99. This indicates very good intraexaminer reliability (Table 1).

**Table 1:** Intra-examiner reliability of measurements of histomorphometric parameters and immunohistochemical analysis

Variable	ICC coefficient	95% C.I
**1st measurements vs. 2nd measurements**	0.994	0.991–0.996

### Demographic data

Twenty male, 8-weeks-old, New Zealand rabbits were used in the current study. Demographic data and sample characteristics are shown in Table 2.

**Table 2:** Demographic data of the sample

Covariate	All sample (***n*** = 20)
Amount of expansion attained mm (T1-T0) Mean ± SD	4.08 ± 0.32
Initial animal weight (kg) Mean ± SD	2.210 ± 0.159
Animal weight before euthanesia (kg) Mean ± SD	3.255 ± 0.201
Animal sex	
Male n (%)	20 (100%)
Female n (%)	0 (0%)
Animal age at start of study (days) Mean ± SD	59.15 ± 2.54

The expansion (difference in distance between mesio-incisal angles of both right and left central incisors at T1 and T0) was successful in all the animals with mean separation of the central incisors teeth 4.11 mm in control group A and 4.06 mm in study group B. All included animals were same age and average weight suitable for this age. No significant diffrences were observed in the animals’ demographic data and the study covariates between the different study groups (Table 3).

**Table 3:** Demographic data distribution in the study groups

	Control (***n*** = 10)	Study (PRP injection) (***n*** = 10)	t	*P*
**Amount of expansion in mm (Distance between mesio-incisal angles of both right and left central incisors T1-T0)** Mean ± SD	4.1 ± 0.28	4.06 ± 0.37	0.2508	0.8048
**Initial animals’ weight (kg)** Mean ± SD	2.240 ± 0.196	2.180 ± 0.114	0.8393	0.4123
**Animals’ weight before euthanasia (kg)** Mean ± SD	3.300 ± 0.221	3.210 ± 0.179	1.0000	0.3306
	Paired t test *P* < 0.0001*			
**Animal sex**				
Male	10 (100%)	10 (100%)		
Female	0 (0%)	0 (0%)		
**Animal age at start of study (days)** Mean ± SD	59.10 ± 2.73	59.20 ± 2.49	0.0857	0.9326

* Statistically significant at *p* ≤ 0.05

*SD* Standard deviation

*t* Student t-test

*P p* value for comparing between the studied groups

Paired t-test showed signifant increase in animal weights in both groups (*p* < 0.0001). The gain in animals’ weight was similar in both the control and the study groups with no significant difference in animals’ weight before euthanasia (3.3 ± 0.22 and 3.210 ± 0.179 respectively, *p* = 0.3306) as presented in Table 3. This indicates that the design of the helical spring used for the expansion as well as the intervention procedures (especially PRP injection) were well tolerated by all the animals in both groups with no adverse effects on feeding habits or general health of the animals. No breakages or losses of the expansion spring were noted in any of the study animals.

### Histologic results

**Fig. 4:**
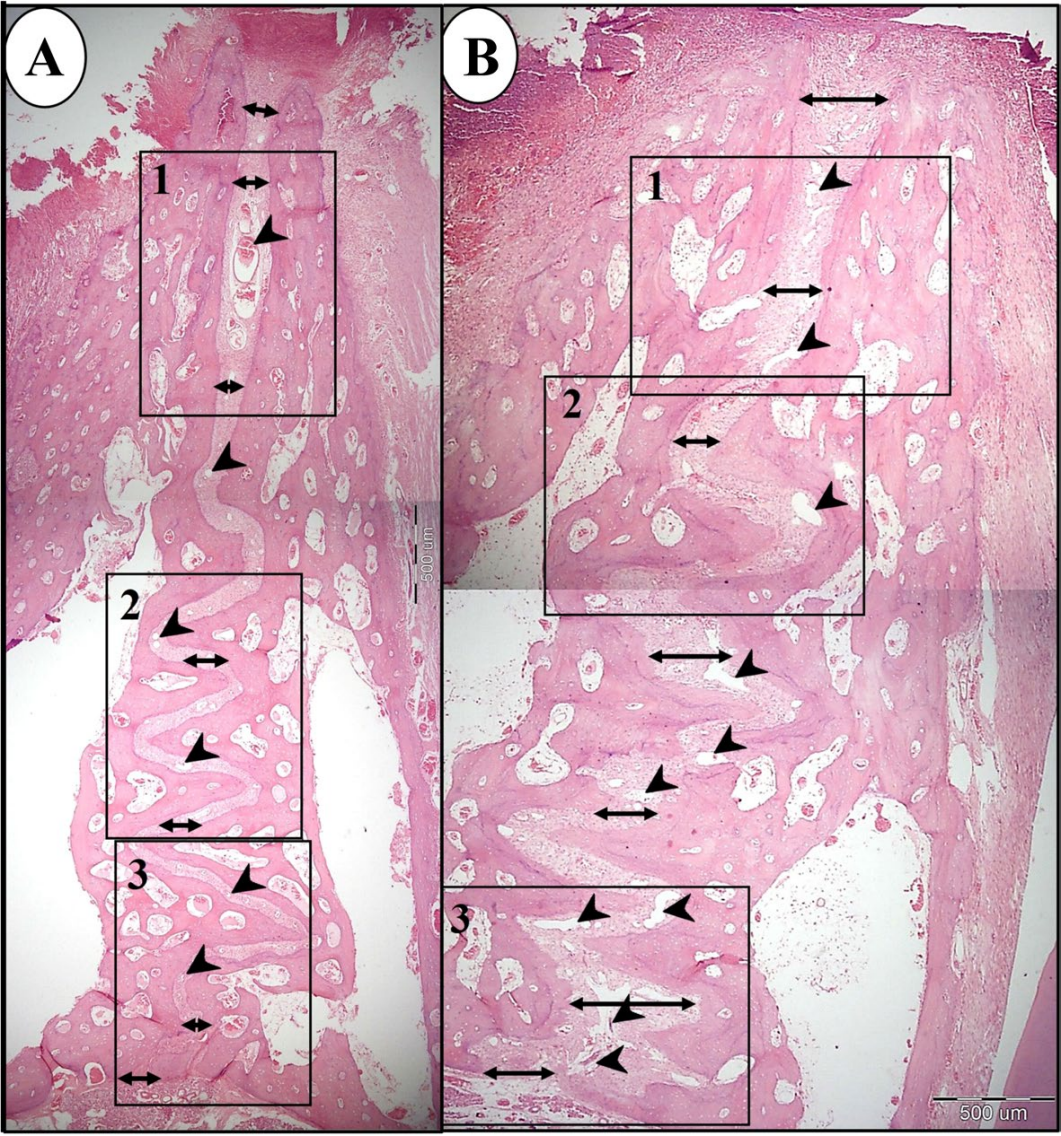
Compound light micrograph (LM) of the mid-palatal suture of control (**a**) and study groups (**b**) showing the greater width of the suture (double headed arrow) in the study group. Also, numerous dilated blood vessels (arrow heads) are seen in study group **B** compared to few scattered blood vessels (arrow heads) in control group **A**. H&E × 40

**Fig. 5:**
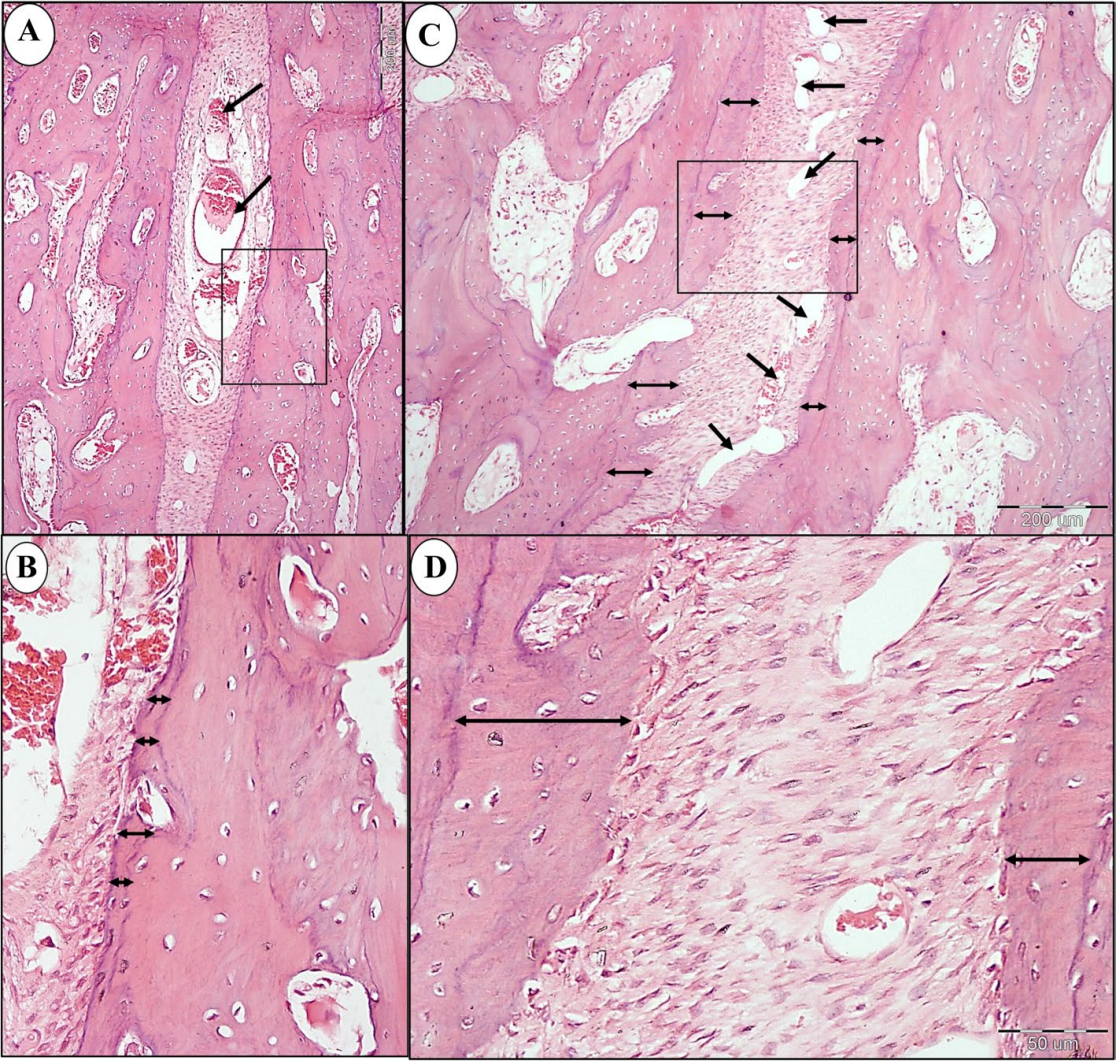
LM of control (A & B) and study groups (C & D) showing the anterior part of the suture. **a** A higher magnification of (inset 1 in Fig. 4a) of control group showing some dilated blood vessels (arrows). **b** A higher magnification of inset in the previous photograph showing the formation of a narrow band of new bone (double headed arrows). **c** A higher magnification of (inset 1 in Fig. 4b) showing the formation of a wider band of new bone (double headed arrows) on each side of the suture. Multiple blood vessels can be seen (arrows). **d** A higher magnification of inset in previous photograph showing the width of the new bone on each side (double headed arrows) of the suture. (H&E; A&C × 100, B&D × 400)

**Fig. 6:**
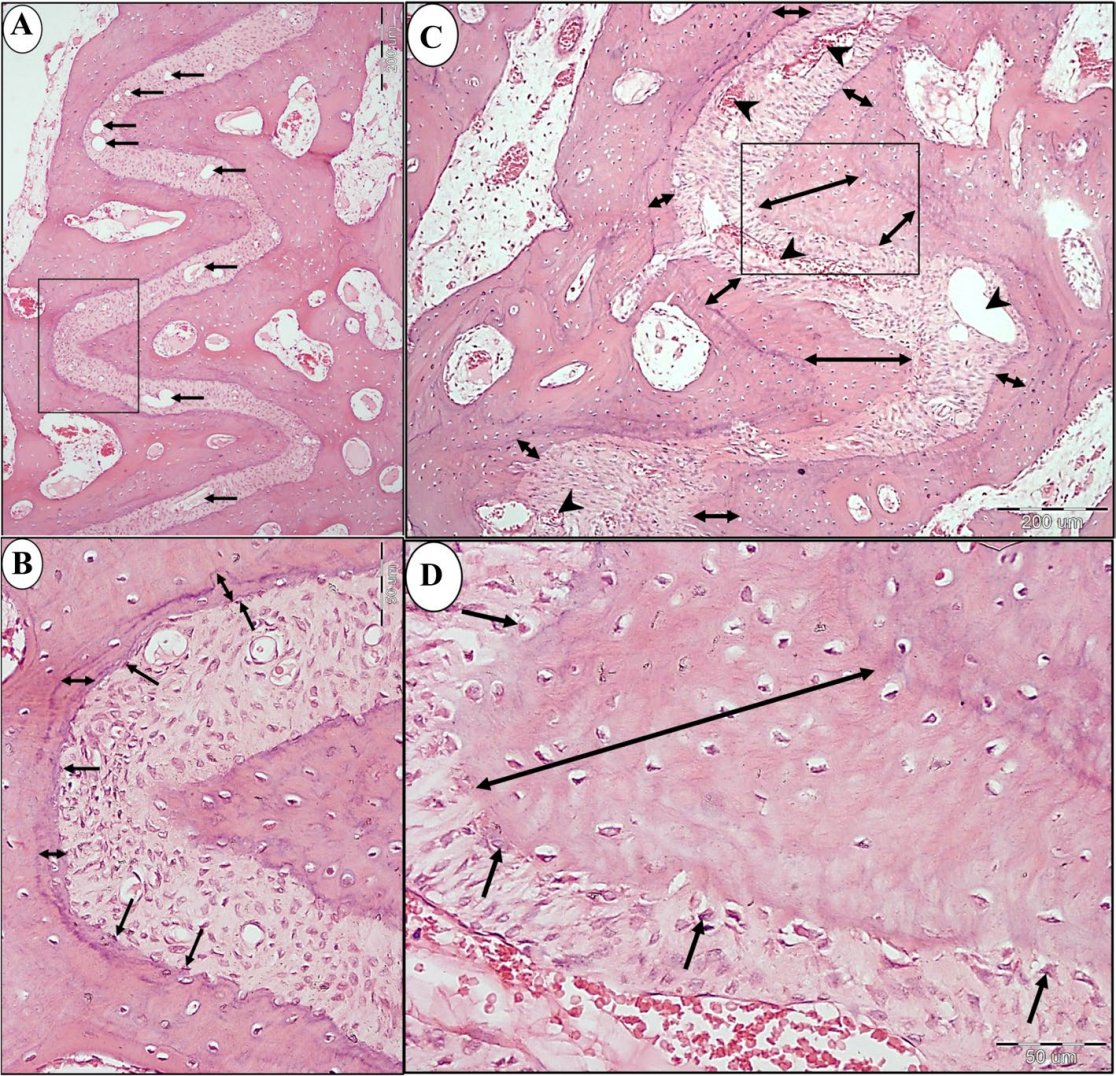
LM of control (a & b) and study groups (C & D) showing the middle part of the suture. **a** A higher magnification of (inset 2 in Fig. 4 a) of control group showing numerous small blood vessels (arrows). **b** A higher magnification of the inset in the previous photograph showing a narrow band of new bone formation on one side of the suture (double headed arrows) and flattened osteoblasts (arrows) lining the bone surface. **c** A higher magnification of (inset 2 in Fig. 4b) of study group showing the formation of a wide band of new bone (double headed arrows) on each side of the suture and numerous dilated blood vessels (arrow heads). **d** A higher magnification of the inset in the previous photograph showing the width of the new bone (double headed arrow) which is covered by voluminous osteoblasts (arrows). (H&E; A&C × 100, B&D × 400)

**Fig. 7:**
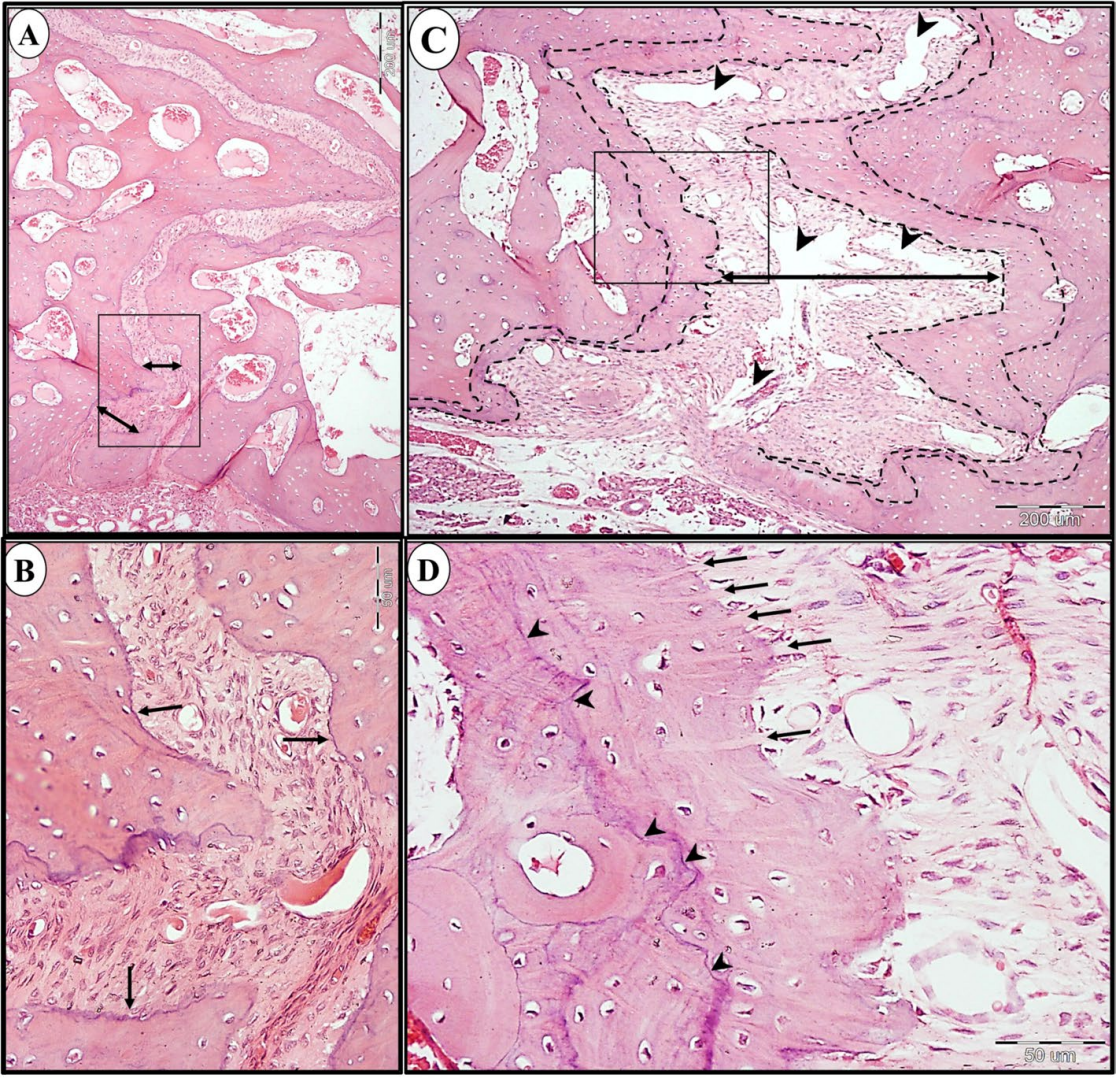
LM of control (a & b) and study groups (C & D) showing the posterior part of the suture. **a** A higher magnification of (inset 3 in Fig. 4a) of control group showing the narrow suture width (double headed arrows). **b** A higher magnification of the inset in the previous photograph showing the almost absence of new bone formation on each side of the suture (arrows). **c** A higher magnification of (inset 3 in Fig. 4b) of study group showing the widely expanded suture (double headed arrow) and the formation of new bone (between the dotted lines) on each side of the suture. Numerous dilated blood vessels are seen (arrow heads). **d** A higher magnification of the inset in the previous photograph showing reversal line (arrow heads) between the old & new bone and the inserted fibers (arrows) into the new bone. (H&E; A&C × 100, B&D × 400)

Light microscopic examination of specimens of control group (A) revealed a narrow suture width along its length with few small blood vessels. Narrow band of new bone was seen at the anterior and middle portions of the suture (Fig. 4a, Fig. 5a, b, Fig. 6a, b). However, almost no bone was formed at the posterior end of the suture (Fig. 7a, b). Flattened osteoblasts were seen covering the bone surface (Fig. 6b). On the other hand, in study group (B), the expanded suture showed greater width with numerous dilated blood vessels were found along the suture length till its posterior end (Fig. 4b, Fig. 5c, Fig. 6c, Fig. 7c). A wide band of newly formed bone was seen on each side of the suture all over its length till its end (Figs. 5c, d, Fig. 6 c, d, Fig. 7c, d) with the presence of newly inserted fibers (Fig. 7d) which indicates the good union of suture fibres with the newly formed bone on each side of the suture. In addition, voluminous osteoblasts were seen covering the bone surface which indicates active bone formation (Fig. 6d).

**Fig. 8:**
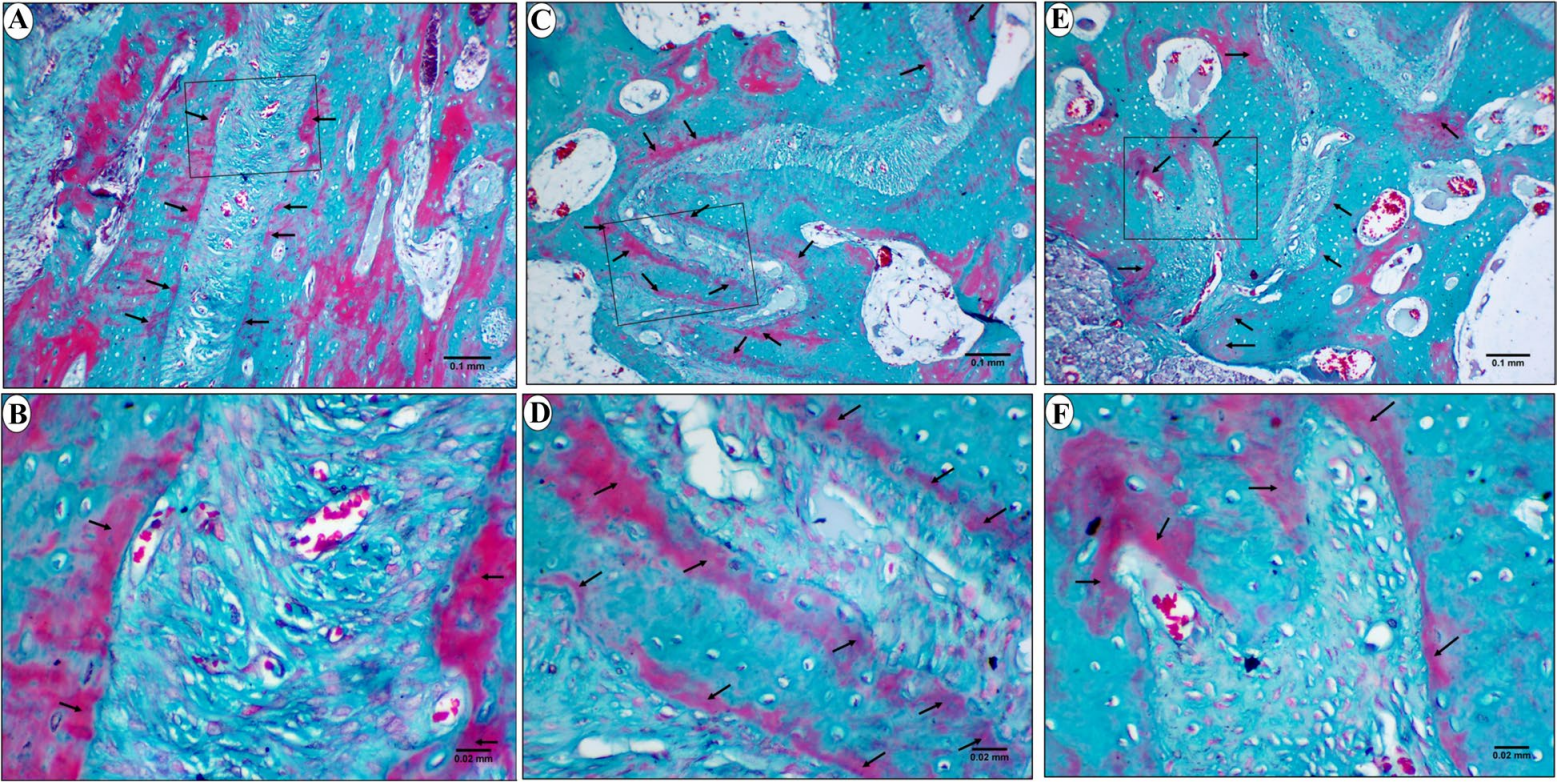
LM of control group showing the anterior (**a** & **b**), middle (**c** & **d**), and posterior area (**e** & **f**) of the mid-palatal suture. The newly formed unmineralized bone is stained red (arrows). b, d & f are higher magnifications of insets in a,c&e respectively. (Goldner Masson trichrome stain; a,c&e × 100, b,d&f × 400)

**Fig. 9:**
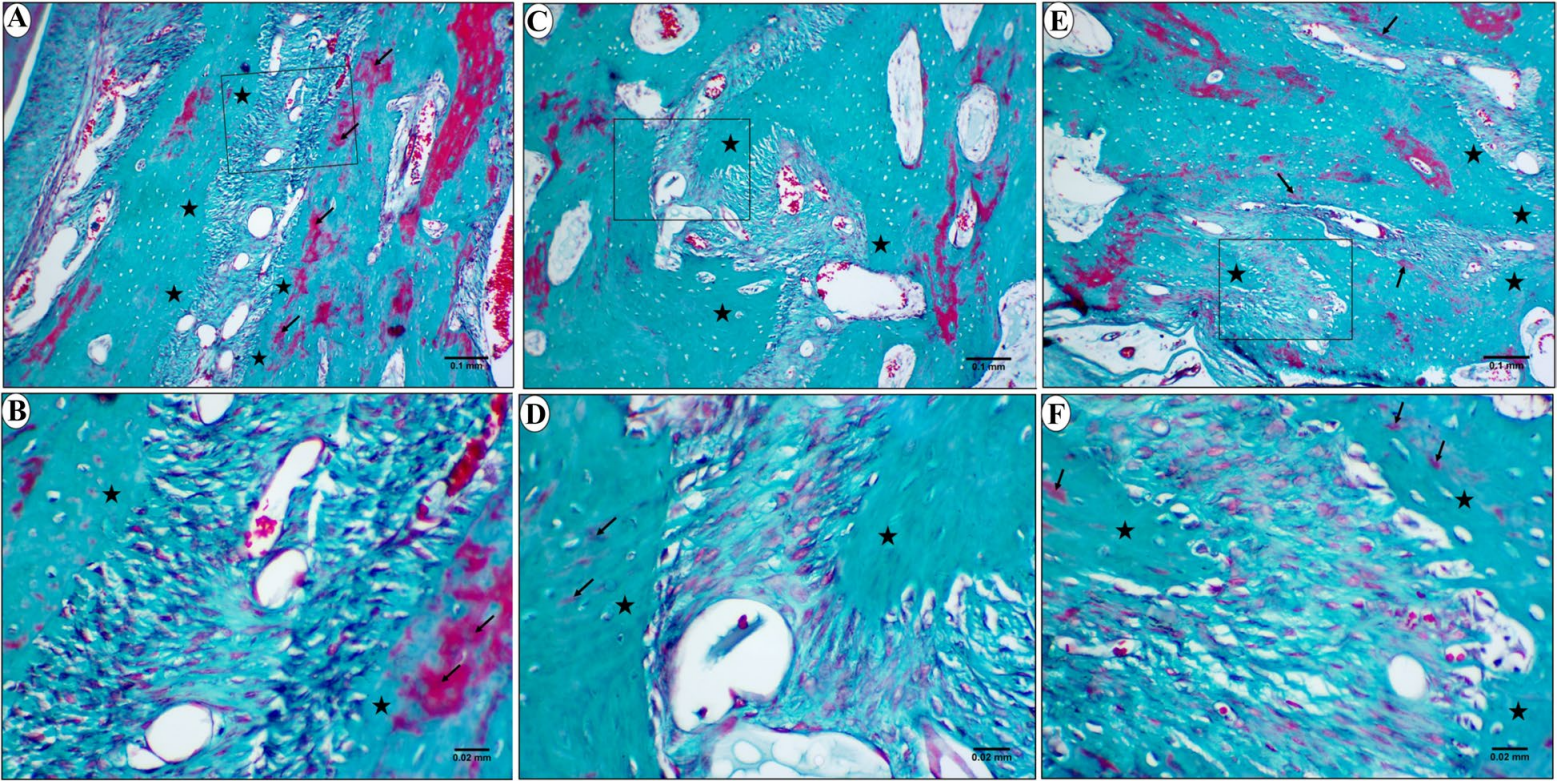
LM of study group showing the anterior (**a** & **b**), middle (**c** & **d**), and posterior area (**e** & **f**) of the mid-palatal suture. Mineralized bone (stars) is stained green with few areas of unmineralized newly formed bone (arrows) are seen in some regions of the suture. b, d & f are higher magnifications of insets in a, c&e respectively. (Goldner Masson trichrome stain; a,c&e × 100, b,d&f × 400)

In Goldner-Masson trichrome stained sections, the control group showed a layer of newly formed unmineralized bone in different areas of the suture (Fig. 8). On the other hand, in study group, most of the newly formed bone was homogenously mineralized all over the suture except for few scattered areas of unmineralized bone (Fig. 9).

### Histomorphometry results

1. **The percentage of new bone surface area.**

**Fig. 10:**
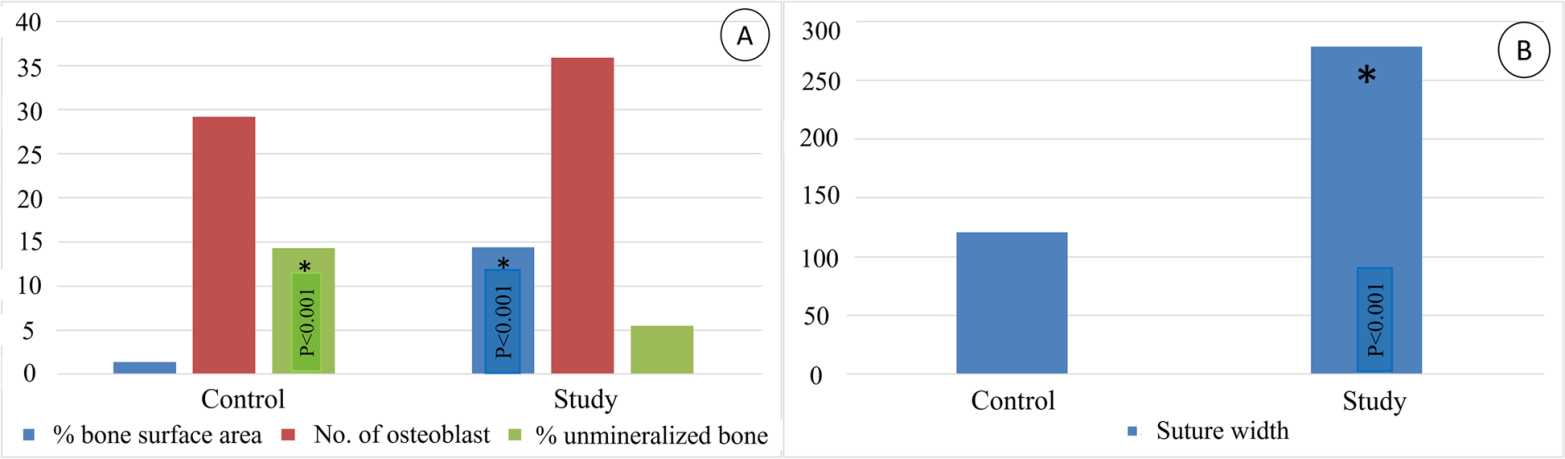
Shows histomorphometrical analysis of (**a**): percentage of surface area of newly formed bone, osteoblasts’ count, and percentage of unmineralized bone. **b**: suture width. (*: Statistically significant at *p* ≤ 0.05)

The percentage of new bone surface area in both groups are shown in Table 4 & Fig. 10a. The study group showed a significant increase in mean percentage of newbone surface area in comparison to control group where the values were 14.4 and 1.4% respectively (*p* < 0.001).

2- **Mean suture width of the mid palatal suture**

**Table 4:** Comparison between the two studied groups according to different histomorphometric parameters

	Control (***n*** = 10)	Study (***n*** = 10)	t	*P*
**Percentage of bone surface area**				
Mean ± SD. Median (Min. – Max.)	1.4 ± 0.22 1.3 (1.1–1.8)	14.4 ± 2.7 15.4 (8.6–17.2)	15.109	< 0.001^*^
**Suture width (μms)**				
Mean ± SD. Median (Min. – Max.)	120.4 ± 3.4 120.6 (115.2–125.4)	278.8 ± 9 277 (267–292.2)	52.188	< 0.001^*^
**No. of osteoblasts**				
Mean ± SD. Median (Min. – Max.)	29.20 ± 12.23 32 (13–46)	35.90 ± 9.45 32.50 (24–50)	1.371	0.187
**Percentage of unmineralized bone**				
Mean ± SD. Median (Min. – Max.)	14.32 ± 5.01 12.72 (9.06–20.92)	5.49 ± 2.93 5.14 (0.98–11.07)	4.810	< 0.001^*^

*SD* Standard deviation: *t* Student t-test

*P p* value for comparing between the studied groups

* Statistically significant at *p* ≤ 0.05

The mean suture width showed a significant increase in study group in comparison to control group where the values were 278.8 μms and 120.4 μms respectively (*p* < 0.001). (Table 4 & Fig. 10b).

3- **Osteoblasts’ count**

No statistical significance(*p* = 0.187) was detected in mean osteoblasts’ count between the 2 groups (35.9 for the study group and 29.2 for the control group) (Table 4) & Fig. 10a.

4- **Percentage of unmineralized bone**

The percentage of unmineralized bone in control group was significantly higher (*p* < 0.001) compared to study group (14.32 ± 5.01 and 5.49 ± 2.93 respectively). (Table 4) & Fig. 10a.

### Immunohistochemical analysis

#### CD34

**Fig. 11:**
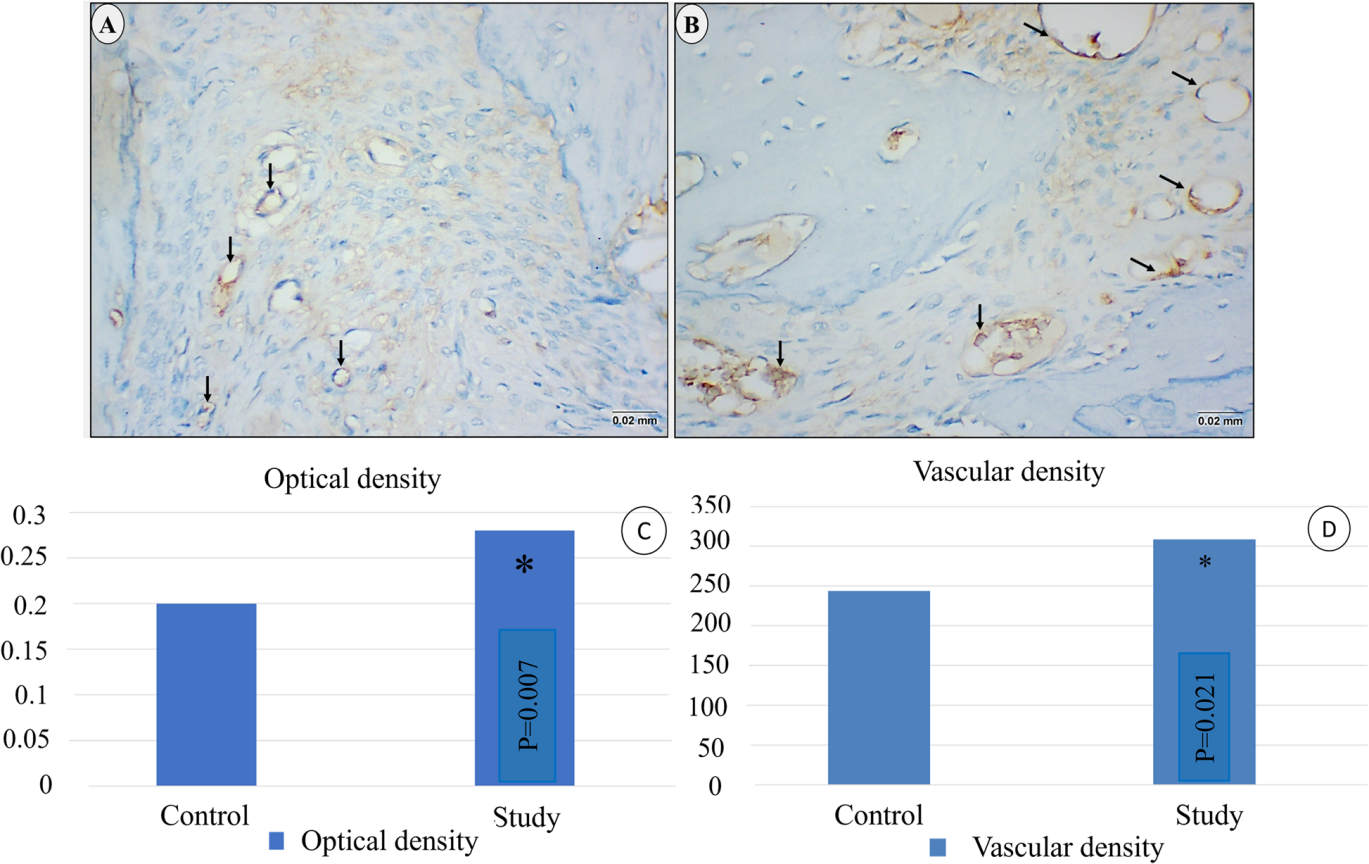
LM of CD34 immuno-staining of blood vessels in control group (**a**) and **s**tudy group (**b**). Study group shows more intense immune-expression of CD34 positive endothelial cells. (× 400) (**c**): Optical density of CD34 immune-staining, (**d**): Vascular density. (* Statistically significant at *p* ≤ 0.05)

Immunohistochemical results revealed that endothelial cells in control group showed faint immunoreactivity to CD34 antibodies (Fig. 11a), while those in the study group revealed stronger immunoreactivity (Fig. 11b).

1. **Optical density**

The optical density of CD34 immuno-reactivity was significantly higher (*p* = 0.007) in study group compared to control group (0.28 and 0.2 respectively). (Table 5) & Fig. 11c.

2- **Vascular density**

**Table 5:** Comparison between the studied groups according to optical density of CD34 immunostaining and vascular density

	Control (***n*** = 10)	Study (***n*** = 10)	t	*P*
**Optical density**				
Mean ± SD.	0.20 ± 0.02	0.28 ± 0.07	3.409	0.007^*^
Median (Min. – Max.)	0.20 (0.17–0.24)	0.29 (0.19–0.39)		
**Vascular density**				
Mean ± SD.	243.86 ± 48.10	309 ± 65.34	2.539	0.021^*^
Median (Min. – Max.)	245.48 (137.93–307.69)	307.88 (200–423.08)		

*SD* Standard deviation: *t* Student t-test

*p p* value for comparing between the studied groups

* Statistically significant at *p* ≤ 0.05

A statistically significant (*p* = 0.021) increase in the number of blood vessels in the study group was found in comparison to the control group; vascular density was 309 ± 65.34/mm^2^ and 243.86 ± 48.1/mm^2^ respectively. (Table 5) & Fig. 11d.

### Osteopontin

**Fig. 12:**
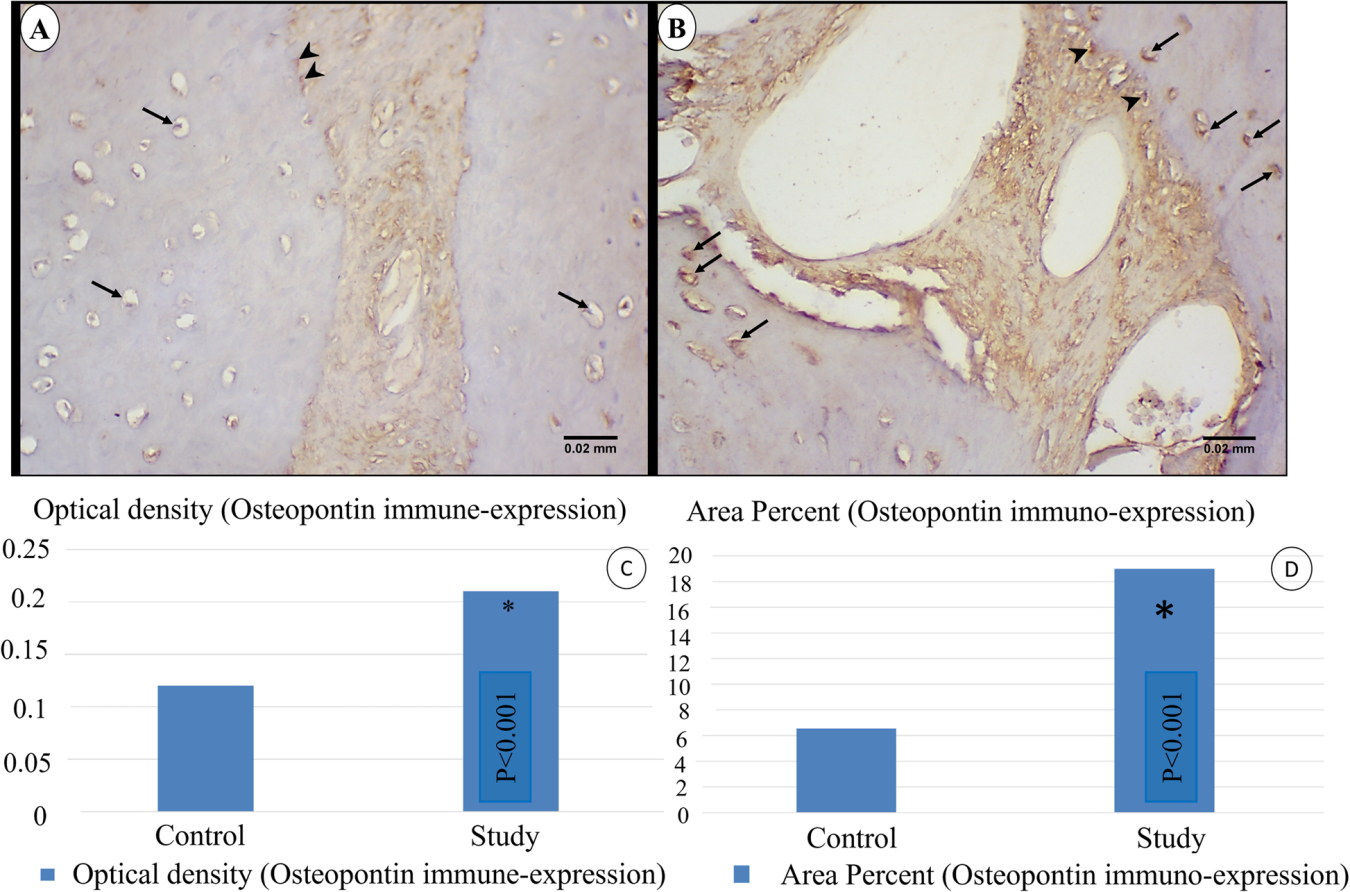
LM of Osteopontin immune-staining in control (**a**) and **s**tudy group (**b**). Study group shows more intense immune-expression of osteopontin in osteoblasts (arrow heads) and osteocytes (arrows). (× 400) (**c**): Optical density of osteopontin immune-staining, (**d**): area percent of osteopontin expression. (* Statistically significant at p ≤ 0.05)

Immunohistochemical analysis showed a stronger immune expression of osteopontin in both osteoblasts and osteocytes in study group compared to control group (Fig. 12a, b).

1. Optical density: showed a statistically significant increase in optical density of immune-reactivity in study group compared to control group (*p* < 0.001), where the values were 0.21 ± 0.02 & 0.12 ± 0.01 respectively (Table 6) & Fig. 12c.
2. Area percent: the ratio of area stained positive to osteopontin to the total area of the field was significantly greater in study group (18.99 ± 2.76) compared to control group (6.56 ± 3.44) (*p* < 0.001) (Table 6) & Fig. 12d.

**Table 6:** Comparison between the two studied groups according to Osteopontin immunostaining

	Control (*n* = 10)	Study (*n* = 10)	t	P
Optical density				
Mean ± SD.	0.12 ± 0.01	0.21 ± 0.02	11.347	< 0.001^*^
Median (Min. – Max.)	0.12 (0.11–0.15)	0.21 (0.16–0.23)		
Area percent				
Mean ± SD.	6.56 ± 3.44	18.99 ± 2.76	8.911	< 0.001^*^
Median (Min. – Max.)	5.43 (2.07–12.54)	19.32 (13.74–23.18)		

*SD* Standard deviation: *t* Student t-test

*p p* value for comparing between the studied groups

* Statistically significant at *p* ≤ 0.05

## Discussion

This randomized controlled animal study was executed to test the hypothesis that a platelet concentrate platelet rich plasma (PRP) can be used as a natural method to induce and accelerate new bone formation in the expanded midpalatal suture. The literature reported some successful attempts for accelerating midpalatal bone formation following midpalatal expansion [15–23]. However, the use of a platelete concentrate would be advantageous over previously tested methods being an autologous product eliminating the risk of cross infections or provoking any adverse reactions [27]. In the current study, only injectable forms of platelet concentrate; PRP or i-PRF could be used and the authors used PRP. The authors used PRP as no scientific evidence supports the absolute superiority of i-PRF over PRP [40, 41, 43, 44] and to avoid the anticipated problems that may be encountered with the use of i-PRF. The success of i-PRF use is dependent on speedy manipulation of the blood sample from collection till injection of the concentrate before the activation of clotting cascade [75]. This introduced inconveniences to the authors as blood samples were collected in animal house and platelet concentrates were prepared in the faculty of medicine central laboratories. Another consideration was the low quantity of i-PRF obtained from a certain volume of blood in comparison to the amount of PRP obtained from the same blood volume [76]. This was of prime importance in this study as the allowable quantity of blood to be withdrawn from each rabbit without adverse health effects is limited.

The histologic results of the current study revealed that in PRP injected group, an even wide band of new bone was seen along the whole length of the suture. On the other hand, the control group showed a narrower band of new bone along the suture bony boundaries. Our results are in agreements with the report by Xu et al. [31] who found that PRP injection resulted in new bone stimulation in the distracted sagittal suture, however, with no signs of suture fusion. In addition, other studies proved that PRP can increase bone formation in critical size defects [77] and fracture in rats [78]. Contrary to our results, a recent study by Ebadifar et al. [79] concluded that PRP alone resulted in an increase in sutural bone density after maxillary expansion, however; the increase was not significant. They found that the combined injection of PRP with mesenchymal stem cells resulted in a significant increase in bone formation.

The positive effect of PRP can be explained by its anabolic effect on bone formation. PRP contains many growth factors including platelet derived growth factor (PDGF), vascular endothelial growth factor (VEGF), transforming growth factor (TGF β1 and β2), insulin-like growth factor (IGF), interleukin-1 (IL-1), platelet-derived angiogenesis factor (PDAF), and platelet-derived endothelial growth factor (PDEGF) [27, 80, 81]. All these growth factors interact with osteoblast cell surface receptors leading to increase in cell proliferation and osteoid formation [82]. Steller et al. [83] found that PRP can increase viability, migration and proliferation of osteoblasts treated with zoledronic acid. Moreover, PRP can increase alkaline phosphatase activity of osteoblasts in a dose-dependent manner [29] and also, it can upregulate osteocalcin and TGF β1 expression and downregulate osteoprotegerin [84].

Our histomorphometric results confirmed the light microscopic results. A statistically significant (*p* < 0.001) increase in percentage of new bone surface area was found in the study group compared to the control group. Our results are consistent with previous studies that also proved the positive effect of PRP on enhancement of bone formation. Oley et al. [85] concluded that PRP with bone graft material can increase bone formation in rats’ cranial bone defect when compared to bone graft alone. Emilov-Velev et al. [86] also proved that PRP in conjunction with calcium phosphate cement resulted in increase in new bone formation than the graft material alone.

Our histomorphometric analysis showed a statistically significant increase in suture width in the study group compared to control group (278.8 & 120.4 μm respectively), where *p* < 0.001. This indicates that PRP injection prevented relapse of expanded mid-palatal suture.

The histomorphometric analysis of the Goldner Masson trichrome stained sections of the current study, revealed a significant increase in the percentage of unmineralized bone in control group compared to study group. This may be due to the role of PRP in enhancement of bone mineralization. PRP stimulates alkaline phosphatase expression in osteoblasts [29, 87] which facilitates mineralization by increasing local concentrations of inorganic phosphate [88].

In the current study, a statistically significant increase in optical density of CD34 immunoreactivity and vascular density in study group compared to control group. Our findings agree with those of Zhang et al. who reported increase in vascular density in CD34 stained sections in rat calvarial bone defect when treated with PRP and autogenous bone graft than those defects treated with bone graft alone [89]. This could be attributed to PRP containing angiogenic factors like VEGF, PDGF and fibroblast growth factor (FGF). VEGF increases angiogenesis, mitosis and migration of endothelial cells, and also enhances permeability of the vessels [90].

Immunohistochemical analysis of our work showed a statistically significant increase in immune-expression of osteopontin in osteoblasts and osteocytes and its area percent in study group than that in control group. Our results are supported by the results of Nagata et al. [91] who found that PRP in conjunction with bone graft increases the expression of osteopontin in bone cells in surgically created bone defect compared to bone graft alone. In addition, Hu et al. [92]. reported that PRP upregulates osteopontin expression and other bone proteins and also increases angiogenesis in vitro.

### Study limitations

Evaluation of bone formation in the expanded midpalatal suture at different time intervals (for example 3 weeks and 6 weeks) would allow evaluation of the effect of PRP injection on sequential histological changes in the expanded midpaltal suture.

## Conclusion

Based on the results of the current study, PRP injection has an anabolic effect on bone and increases bone vascularity in the expanded mid-palatal suture in rabbits, and subsequently can stimulate new bone formation to a great extent. This could hopefully have a positive effect on expansion stability and the required post expansion retention period. However, further research to study the effect of enhanced bone formation in the expanded midpalatal suture on expansion stability and post expansion retention period is recommended.

## Data Availability

The datasets used and analysed during the current study are available from the corresponding author on reasonable request.

## Abbreviations

PRP: Platelet Rich Plasma
H&E: Hematoxylin and Eosin stain
OD: Optical density
ICC: Intraclass Correlation Coefficient
PBS: Phosphate Buffered Saline
PDGF: Platelet Derived Growth Factor
VEGF: Vascular Endothelial Growth Factor
TGF: Transforming Growth Factor
IGF: Insulin-Like Growth Factor
IL-1: Interleukin-1
PDAF: Platelet-Derived Angiogenesis Factor
PDEGF: Platelet-Derived Endothelial Growth Factor

## References

[1] Kobayashi ET, Hashimoto F, Kobayashi Y, Sakai E, Miyazaki Y, Kamiya T (1999). Force-induced rapid changes in cell fate at midpalatal suture cartilage of growing rats. J Dent Res.

[2] Katebi N, Kolpakova-Hart E, Lin CY, Olsen BR (2012). The mouse palate and its cellular responses to midpalatal suture expansion forces. Orthod Craniofacial Res.

[3] Cameron CG, Franchi L, Baccetti T, McNamara JA (2002). Long-term effects of rapid maxillary expansion: a posteroanterior cephalometric evaluation. Am J Orthod Dentofac Orthop.

[4] Lima AL, Lima Filho RMA, Bolognese AM (2005). Long-term clinical outcome of rapid maxillary expansion as the only treatment performed in class I malocclusion. Angle Orthod.

[5] Sannomiya EK, Macedo MMC, Siqueira DF, Goldenberg FC, Bommarito S (2007). Evaluation of optical density of the midpalatal suture 3 months after surgically assisted rapid maxillary expansion. Dentomaxillofac Radiol.

[6] Alyessary AS, Yap AU, Othman SA, Rahman MT, AL-namnam NM, Radzi Z (2018). Is there an optimal initial amount of activation for midpalatal suture expansion?: A histomorphometric and immunohistochemical study in a rabbit model. J Orofac Orthop.

[7] Willershausen I, Erbe C, Al-Maawi S, Orlowska A, Wehrbein H, Ghanaati S (2019). Development of a novel histological and histomorphometric evaluation protocol for a standardized description of the mid-palatal suture - an ex vivo study. J Anat.

[8] Lin G, Finger E, Gutierrez-Ramos JC (1995). Expression of CD34 in endothelial cells, hematopoietic progenitors and nervous cells in fetal and adult mouse tissues. Eur J Immunol.

[9] Wang KX, Denhardt DT (2008). Osteopontin: role in immune regulation and stress responses. Cytokine Growth Factor Rev.

[10] Si J, Wang C, Zhang D, Wang B, Zhou Y (2020). Osteopontin in bone metabolism and bone diseases. Med Sci Monit Int Med J Exp Clin Res.

[11] Terai K, Takano-Yamamoto T, Ohba Y, Hiura K, Sugimoto M, Sato M (1999). Role of osteopontin in bone remodeling caused by mechanical stress. J Bone Miner Res.

[12] Miles RR, Turner CH, Santerre R, Tu Y, McClelland P, Argot J (1998). Analysis of differential gene expression in rat tibia after an osteogenic stimulus in vivo: mechanical loading regulates osteopontin and myeloperoxidase. J Cell Biochem.

[13] You J, Reilly GC, Zhen X, Yellowley CE, Chen Q, Donahue HJ (2001). Osteopontin gene regulation by oscillatory fluid flow via intracellular calcium mobilization and activation of mitogen-activated protein kinase in MC3T3-E1 osteoblasts. J Biol Chem.

[14] Perrien DS, Brown EC, Aronson J, Skinner RA, Montague DC, Badger TM (2002). Immunohistochemical study of osteopontin expression during distraction osteogenesis in the rat. J Histochem Cytochem Off J Histochem Soc.

[15] Saito S, Shimizu N (1997). Stimulatory effects of low-power laser irradiation on bone regeneration in midpalatal suture during expansion in the rat. Am J Orthod Dentofac Orthop.

[16] Angeletti P, Pereira MD, Gomes HC, Hino CT, Ferreira LM. Effect of low-level laser therapy (GaAlAs) on bone regeneration in midpalatal anterior suture after surgically assisted rapid maxillary expansion. Oral Surg Oral Med Oral Pathol Oral Radiol Endod. 2010;109(3) 10.1016/j.tripleo.2009.10.043 20219584

[17] Tang GH, Xu J, Chen RJ, Qian YF, Shen G (2011). Lithium delivery enhances bone growth during midpalatal expansion. J Dent Res.

[18] Uysal T, Amasyali M, Olmez H, Enhos S, Karslioglu Y, Gunhan O (2011). Effect of vitamin C on bone formation in the expanded inter-premaxillary suture. Early bone changes. J Orofac Orthop.

[19] Uysal T, Amasyali M, Enhos S, Sonmez MF, Sagdic D (2009). Effect of ED-71, a new active vitamin D analog, on bone formation in an orthopedically expanded suture in rats. A Histomorphometric Study. Eur J Dent.

[20] Oztürk F, Babacan H, Inan S, Gümüş C (2011). Effects of bisphosphonates on sutural bone formation and relapse: A histologic and immunohistochemical study. Am J Orthod Dentofac Orthop.

[21] Uysal T, Ustdal A, Sonmez MF, Ozturk F (2009). Stimulation of bone formation by dietary boron in an orthopedically expanded suture in rabbits. Angle Orthod.

[22] Kara MI, Erciyas K, Altan AB, Ozkut M, Ay S, Inan S (2012). Thymoquinone accelerates new bone formation in the rapid maxillary expansion procedure. Arch Oral Biol.

[23] Ozdemir H, Kara MI, Erciyas K, Ozer H, Ay S (2012). Preventive effects of thymoquinone in a rat periodontitis model: a morphometric and histopathological study. J Periodontal Res.

[24] Babbush CA, Kevy SV, Jacobson MS (2003). An in vitro and in vivo evaluation of autologous platelet concentrate in oral reconstruction. Implant Dent.

[25] Eppley BL, Woodell JE, Higgins J (2004). Platelet quantification and growth factor analysis from platelet-rich plasma: implications for wound healing. Plast Reconstr Surg.

[26] Fréchette J-P, Martineau I, Gagnon G (2005). Platelet-rich plasmas: growth factor content and roles in wound healing. J Dent Res.

[27] Marx RE, Carlson ER, Eichstaedt RM, Schimmele SR, Strauss JE, Georgeff KR (1998). Platelet-rich plasma: growth factor enhancement for bone grafts. Oral Surg Oral Med Oral Pathol Oral Radiol Endod.

[28] Kassolis JDJ, Reynolds MA (2005). Evaluation of the adjunctive benefits of platelet-rich plasma in subantral sinus augmentation. J Craniofac Surg.

[29] Herrera BS, Coimbra LS, Bastos AS, Teixeira SA, Steffens JP, Muscara MN (2012). Platelet-rich plasma stimulates cytokine expression and alkaline phosphatase activity in osteoblast-derived osteosarcoma cells. Arch Oral Biol.

[30] Zhong W, Sumita Y, Ohba S, Kawasaki T, Nagai K, Ma G (2012). In vivo comparison of the bone regeneration capability of human bone marrow concentrates vs. platelet-rich plasma. PLoS One.

[31] Xu H, Ke K, Zhang Z, Luo X, Jin X, Liu SS-Y (2013). Effects of platelet-rich plasma and recombinant human bone morphogenetic protein-2 on suture distraction osteogenesis. J Craniofac Surg.

[32] Fujioka-Kobayashi M, Miron RJ, Hernandez M, Kandalam U, Zhang Y, Choukroun J (2017). Optimized Platelet-rich fibrin with the low-speed concept: growth factor release, biocompatibility, and cellular response. J Periodontol.

[33] Miron RJ, Fujioka-Kobayashi M, Hernandez M, Kandalam U, Zhang Y, Ghanaati S (2017). Injectable platelet rich fibrin (i-PRF): opportunities in regenerative dentistry?. Clin Oral Investig.

[34] Choukroun J, Adda F, Schoeffler C, Vervelle A. Une opportunite’ en paro-implantologie: Le PRF. In 2001.

[35] Chang Y-C, Zhao J-H (2011). Effects of platelet-rich fibrin on human periodontal ligament fibroblasts and application for periodontal infrabony defects. Aust Dent J.

[36] Toffler M, Toscano N, Holtzclaw D, Corso M, Ehrenfest DM (2009). Introducing Choukroun’s platelet rich fibrin (PRF) to the reconstructive surgery milieu. J Implant Adv Clin Dent.

[37] Del Corso M, Vervelle A, Simonpieri A, Jimbo R, Inchingolo F, Sammartino G (2012). Current knowledge and perspectives for the use of platelet-rich plasma (PRP) and platelet-rich fibrin (PRF) in oral and maxillofacial surgery part 1: periodontal and dentoalveolar surgery. Curr Pharm Biotechnol.

[38] Choukroun J, Diss A, Simonpieri A, Girard M-O, Schoeffler C, Dohan SL (2006). Platelet-rich fibrin (PRF): a second-generation platelet concentrate. Part IV: clinical effects on tissue healing. Oral Surg Oral Med Oral Pathol Oral Radiol Endod.

[39] Diab NAF, Ibrahim A-SM, Abdallah AM (2023). Fluid Platelet-rich fibrin (PRF) versus Platelet-rich plasma (PRP) in the treatment of atrophic acne scars: A comparative study. Arch Dermatol Res.

[40] Ammar AM, Al-Sabbagh R, Hajeer MY. Evaluation of the effectiveness of the platelet-rich plasma compared to the injectable platelet-rich fibrin on the rate of maxillary canine retraction: a three-arm randomized controlled trial. Eur J Orthod. 2023;cjad056 10.1093/ejo/cjad056 37796117

[41] Hassan TF, Khaled F, Abdelrahman N, AMR A (2023). Evaluation of the effect of platelet-rich plasma versus platelet-rich fibrin on the rate of tooth movement during alignment of mandibular anterior crowding. Egypt Dent J.

[42] Yao K, Wu Y, Cai J, Wang Y, Shen Y, Jing D (2022). The effect of platelet-rich concentrates on orthodontic tooth movement: A review of randomized controlled trials. Heliyon.

[43] Savva LC (2022). What recent evidence exists to support the use of platelet-rich fibrin in clinical dentistry? A systematic literature review. Oral Surg.

[44] Li Y, Li T, Li J, Tang X, Li R, Xiong Y (2022). Platelet-rich plasma has better results for Retear rate, pain, and outcome than Platelet-rich fibrin after rotator cuff repair: A systematic review and Meta-analysis of randomized controlled trials. Arthrosc J Arthrosc Relat Surg.

[45] Rodriguez IA, Growney Kalaf EA, Bowlin GL, Sell SA (2014). Platelet-rich plasma in bone regeneration: engineering the delivery for improved clinical efficacy. Biomed Res Int.

[46] Harmon K, Hanson R, Bowen J, Greenberg S, Magaziner E, Vandenbosch J, et al. Guidelines for the use of Platelet rich plasma. Int Cell Med Soc. 2012;

[47] Andrews RK, Berndt MC (2004). Platelet physiology and thrombosis. Thromb Res.

[48] Choukroun J. Advanced PRF &i-PRF: platelet concentrates or blood concentrates? J Periodont Med Clin Pr. 2014;1

[49] Serafini G, Lopreiato M, Lollobrigida M, Lamazza L, Mazzucchi G, Fortunato L (2020). Platelet rich fibrin (PRF) and its related products: biomolecular characterization of the liquid fibrinogen. J Clin Med.

[50] Tischler M (2002). Platelet rich plasma. Utilizing autologous growth factors for dental surgery to enhance bone and soft tissue grafts. NY State Dent J.

[51] Anila S, Nandakumar K (2006). Applications of platelet rich plasma for regenerative therapy in periodontics. Trends Biomater Artif Organs.

[52] Jain S, Bunkar A (2020). Overview of platelet-rich plasma: orthodontics perspective. Int J Contemp Dent Med Rev.

[53] Güleç A, Bakkalbaşı BÇ, Cumbul A, Uslu Ü, Alev B, Yarat A (2017). Effects of local platelet-rich plasma injection on the rate of orthodontic tooth movement in a rat model: A histomorphometric study. Am J Orthod Dentofac Orthop.

[54] Liu L, Kuang Q, Zhou J, Long H (2021). Is platelet-rich plasma able to accelerate orthodontic tooth movement?. Evid Based Dent.

[55] Abdel-Haffiez SH, Ismail HI, Elharouni NM, Ali HM (2017). The effect of platelet rich plasma injection on relapse of orthodontically moved teeth in rabbits. Egypt Orthod J.

[56] Efeoglu C, Akçay YD, Ertürk S (2004). A modified method for preparing platelet-rich plasma: an experimental study. J Oral Maxillofac Surg.

[57] Nunamaker DM. Experimental models of fracture repair. Clin Orthop Relat Res. 1998;355(355 Suppl) 10.1097/00003086-199810001-00007 9917626

[58] Charan J, Biswas T (2013). How to calculate sample size for different study designs in medical research?. Indian J Psychol Med.

[59] Pannucci CJ, Wilkins EG (2010). Identifying and avoiding bias in research. Plast Reconstr Surg.

[60] Faul F, Erdfelder E, Lang AG, Buchner A (2007). G*power 3: a flexible statistical power analysis program for the social, behavioral, and biomedical sciences. Behav Res Methods.

[61] Percie du Sert N, Hurst V, Ahluwalia A, Alam S, Avey MT, Baker M (2020). The ARRIVE guidelines 2.0: updated guidelines for reporting animal research. J Cereb blood flow Metab Off J Int Soc Cereb Blood Flow Metab.

[62] Roche JJ, Cisneros GJ, Acs G (1997). The effect of acetaminophen on tooth movement in rabbits. Angle Orthod.

[63] Drevensek M, Sprogar S, Boras I, Drevensek G (2006). Effects of endothelin antagonist tezosentan on orthodontic tooth movement in rats. Am J Orthod Dentofac Orthop.

[64] Suresh K (2011). An overview of randomization techniques: an unbiased assessment of outcome in clinical research. J Hum Reprod Sci.

[65] Nishioka T, Yokota M, Tsuda I, Tatsumi N (2002). Flow cytometric analysis of platelet activation under calcium ion-chelating conditions. Clin Lab Haematol.

[66] Nagata MJH, Messora MR, Furlaneto FAC, Fucini SE, Bosco AF, Garcia VG (2010). Effectiveness of two methods for preparation of autologous platelet-rich plasma: an experimental study in rabbits. Eur J Dent.

[67] Luengo Gimeno F, Gatto S, Ferro J, Croxatto JO, Gallo JE (2006). Preparation of platelet-rich plasma as a tissue adhesive for experimental transplantation in rabbits. Thromb J.

[68] MacNeill SR, Cobb CM, Rapley JW, Glaros AG, Spencer P (1999). In vivo comparison of synthetic osseous graft materials. A preliminary study. J Clin Periodontol.

[69] Feldman AT, Wolfe D (2014). Tissue processing and hematoxylin and eosin staining. Methods Mol Biol.

[70] Shi Z, Zhong Q, Chen Y, Gao J, Pan X, Lian Q (2021). Nanohydroxyapatite, Nanosilicate-reinforced injectable, and biomimetic gelatin-Methacryloyl hydrogel for bone tissue engineering. Int J Nanomedicine.

[71] Gorustovich AA, Steimetz T, Nielsen FH, Guglielmotti MB (2008). Histomorphometric study of alveolar bone healing in rats fed a boron-deficient diet. Anat Rec (Hoboken).

[72] Egan KP, Brennan TA, Pignolo RJ (2012). Bone histomorphometry using free and commonly available software. Histopathology..

[73] Eslamian L, Ebadifar A, Rad MM, Motamedian SR, Badiee MR, Mohammad-Rahimi H (2020). Comparison of single and multiple low-level laser applications after rapid palatal expansion on bone regeneration in rats. J lasers Med Sci.

[74] Mustafa HN, El Awdan SA, Hegazy GA, Abdel Jaleel GA (2015). Prophylactic role of coenzyme Q10 and Cynara scolymus L on doxorubicin-induced toxicity in rats: biochemical and immunohistochemical study. Indian J Pharm.

[75] Mohan SP, Jaishangar N, Devy S, Narayanan A, Cherian D, Madhavan SS (2019). Platelet-rich plasma and Platelet-rich fibrin in periodontal regeneration: A review. J Pharm Bioallied Sci.

[76] Barhate U, Mangaraj M, Jena A, Sharan J, Trends SJ (2021). Applications of Platelet rich fibrin in dental surgery: A comprehensive literature review. Trends biomater. Artif Organs.

[77] Kim ES, Kim JJ, Park EJ (2010). Angiogenic factor-enriched platelet-rich plasma enhances in vivo bone formation around alloplastic graft material. J Adv Prosthodont.

[78] Guzel Y, Karalezli N, Bilge O, Kacira BK, Esen H, Karadag H (2015). The biomechanical and histological effects of platelet-rich plasma on fracture healing. Knee Surg Sports Traumatol Arthrosc.

[79] Ebadifar A, Eslamian L, Motamedian SR, Badiee MR, Mohaghegh S, Farahani M (2022). Effect of mesenchymal stem cells with platelet-rich plasma carriers on bone formation after rapid maxillary expansion: an animal study. Orthod Craniofacial Res.

[80] Sánchez AR, Sheridan PJ, Kupp LI (2003). Is platelet-rich plasma the perfect enhancement factor? A current review. Int J Oral Maxillofac Implants.

[81] Marx RE (2001). Platelet-rich plasma (PRP): what is PRP and what is not PRP?. Implant Dent.

[82] Schilephake H (2002). Bone growth factors in maxillofacial skeletal reconstruction. Int J Oral Maxillofac Surg.

[83] Steller D, Herbst N, Pries R, Juhl D, Hakim SG. Positive impact of Platelet-rich plasma and Platelet-rich fibrin on viability, migration and proliferation of osteoblasts and fibroblasts treated with zoledronic acid. Sci Rep. 2019;9(1) 10.1038/s41598-019-43798-z PMC6549154 31165745

[84] Graziani F, Ivanovski S, Cei S, Ducci F, Tonetti M, Gabriele M (2006). The in vitro effect of different PRP concentrations on osteoblasts and fibroblasts. Clin Oral Implants Res.

[85] Oley MC, Islam AA, Hatta M, Hardjo M, Nirmalasari L, Rendy L (2018). Effects of platelet-rich plasma and carbonated hydroxyapatite combination on cranial defect bone regeneration: an animal study. Wound Med.

[86] Emilov-Velev K, Clemente-de-Arriba C, Alobera-García MÁ, Moreno-Sansalvador EM, Campo-Loarte J (2015). Bone regeneration in experimental animals using calcium phosphate cement combined with platelet growth factors and human growth hormone. Rev Esp Cir Ortop Traumatol.

[87] Yamakawa J, Hashimoto J, Takano M, Takagi M (2017). The bone regeneration using bone marrow stromal cells with moderate concentration Platelet-rich plasma in femoral segmental defect of rats. Open Orthop J.

[88] Vimalraj S (2020). Alkaline phosphatase: structure, expression and its function in bone mineralization. Gene..

[89] Zhang Z, Zheng Y, Zu J, Zhuang J, Xu G, Yan J (2021). Stromal cell-derived factor (SDF)-1α and platelet-rich plasma enhance bone regeneration and angiogenesis simultaneously in situ in rabbit calvaria. J Mater Sci Mater Med.

[90] Pavlovic V, Ciric M, Jovanovic V, Stojanovic P (2016). Platelet rich plasma: a short overview of certain bioactive components. Open Med.

[91] Nagata M, Messora M, Okamoto R, Campos N, Pola N, Esper L (2009). Influence of the proportion of particulate autogenous bone graft/platelet-rich plasma on bone healing in critical-size defects: an immunohistochemical analysis in rat calvaria. Bone.

[92] Hu Z-M, Peel SAF, Ho SKC, Sándor GKB, Clokie CML (2009). Comparison of platelet-rich plasma, bovine BMP, and rhBMP-4 on bone matrix protein expression in vitro. Growth Factors.

